# Matrine inhibits itching by lowering the activity of calcium channel

**DOI:** 10.1038/s41598-018-28661-x

**Published:** 2018-07-27

**Authors:** Xiao Geng, Hao Shi, Fan Ye, Han Du, Linnan Qian, Leying Gu, Guanyi Wu, Chan Zhu, Yan Yang, Changming Wang, Yuan Zhou, Guang Yu, Qin Liu, Xinzhong Dong, Lei Yu, Zongxiang Tang

**Affiliations:** 1 State Key Laboratory Cultivation Base for TCM Quality and Efficacy, School of Medicine and Life Sciences, Nanjing University of Chinese Medicine, 138 Xianlin Road, Nanjing, 210023 China; 2 MOE Key Laboratory of Model Animal for Disease Study, Model Animal Research Center, Nanjing Biomedical Research Institute, Nanjing University, Nanjing, 210061 China; 3 Department of Anesthesiology and the Center for the Study of Itch, Department of Ophthalmology and Visual Sciences, Washington University School of Medicine, St Louis, MO 63110 USA; 4 The Solomon H. Snyder Department of Neuroscience, Center for Sensory Biology, School of Medicine, Johns Hopkins University, Baltimore, MD 21205 USA; 5 Howard Hughes Medical Institute, School of Medicine, Johns Hopkins University, Baltimore, MD 21205 USA

## Abstract

*Sophorae Flavescentis Radix* (*SFR*) is a medicinal herb with many functions that are involved in anti-inflammation, antinociception, and anticancer. *SFR* is also used to treat a variety of itching diseases. Matrine (MT) is one of the main constituents in SFR and also has the effect of relieving itching, but the antipruritic mechanism is still unclear. Here, we investigated the effect of MT on anti-pruritus. In acute and chronic itch models, MT significantly inhibited the scratching behavior not only in acute itching induced by histamine (His), chloroquine (CQ) and compound 48/80 with a dose-depended manner, but also in the chronic pruritus models of atopic dermatitis (AD) and acetone-ether-water (AEW) in mice. Furthermore, MT could be detected in the blood after intraperitoneal injection (i.p.) and subcutaneous injection (s.c.). Finally, electrophysiological and calcium imaging results showed that MT inhibited the excitatory synaptic transmission from dorsal root ganglion (DRG) to the dorsal horn of the spinal cord by suppressing the presynaptic N-type calcium channel. Taken together, we believe that MT is a novel drug candidate in treating pruritus diseases, especially for histamine-independent and chronic pruritus, which might be attributed to inhibition of the presynaptic N-type calcium channel.

## Introduction

Itch, an unpleasant sensation that evokes a desire to scratch^1^, is one of the important physiological functions that humans and animals acquired during their long-term evolution. It includes acute and chronic itch; acute itch serves us well in guarding against environmental threats^2^; however, chronic pruritus is a burdensome illness that deeply impairs the life quality of millions every year^3^. Inflammatory factors, neuropathic injury, mental disorders and other diseases can cause different degrees of pruritus^4–6^. Lupus erythematosus, pityriasis lichenoides, psoriasis and atopic dermatitis (AD)-induced itching are associated with immune factors produced by the immune system^7–10^. Postherpetic neuralgia (PHN), keloid scars, depression, anxiety, obsessive compulsive disorder, etc., are closely related to neuropathology and psychogenic pruritus^11–14^. Patients with end-stage chronic kidney disease, biliary cirrhosis and sclerosing cholangitis experience pruritus^15,16^. Recently, some new itch receptors have also been discovered. MrgprA3, MrgprC11 and MrgprD are expressed on the DRG neurons of the peripheral nervous system and feel the irritation of peripheral itching^17–19^. MrgprB2 is expressed only in mast cells and is involved in the formation of itch^20^. In the central nervous system, the gastrin-releasing peptide receptor (GRPR) plays an important role in mediating itch sensation^21^. These studies have led to a better understanding of the mechanisms of itch formation, but when we are faced with many patients who are suffering from itching and who feel helpless, new treatments and antipruritic drugs, including traditional Chinese herbal medicines, are becoming a strong desire and an urgent need. More and more researchers are also engaging in the field and trying to find more effective treatment methods and antipruritic drugs.

**Figure 1:**
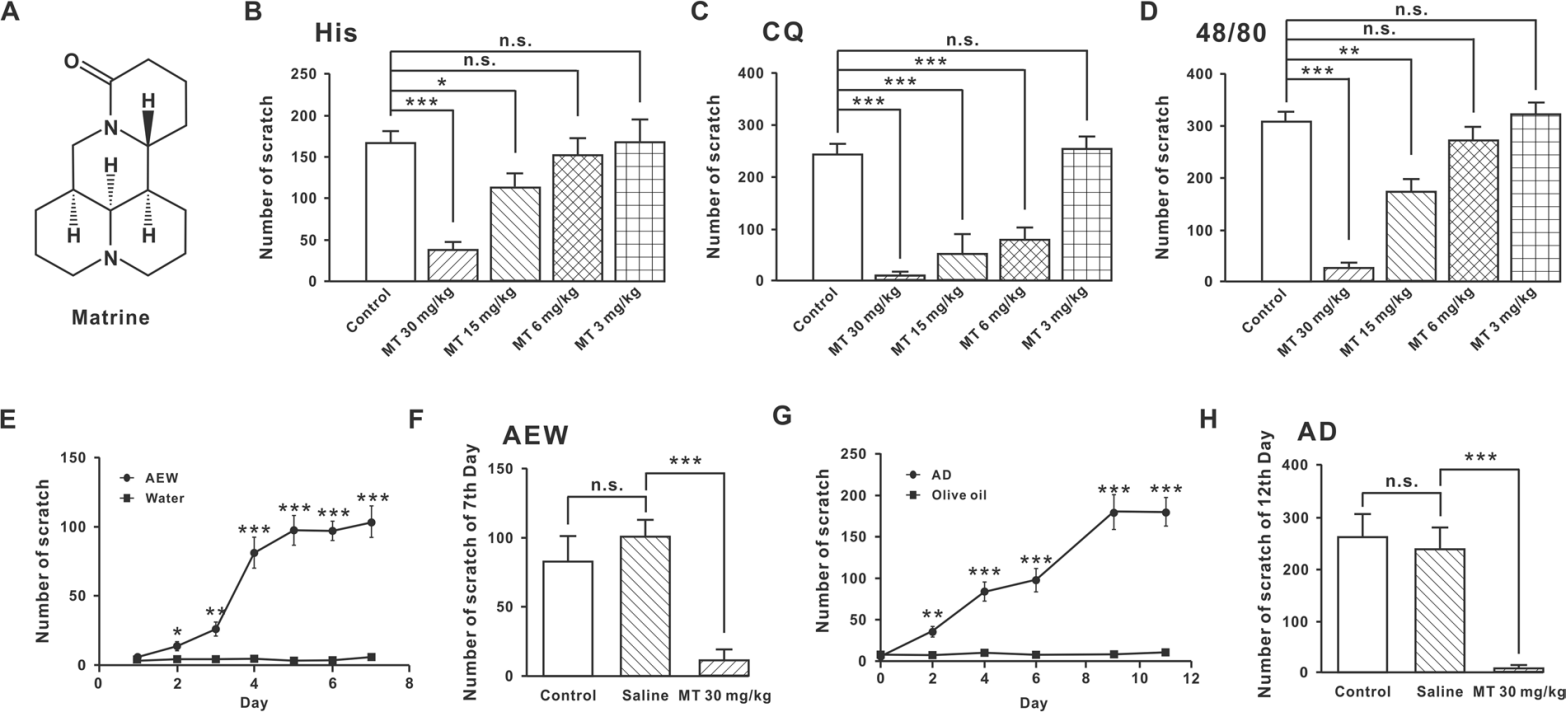
MT could inhibit acute and chronic pruritus. (**A**) Chemical structure of MT. (**B**–**D**) Dose-dependently inhibiting effects of MT (s.c.) on scratching bouts induced by 200 mM histamine, 8 mM chloroquine and 13 mM compound 48/80. (**B**) n = 5 in each group except n = 6 in 6 mg/kg MT group. (**C**) n = 7 in control and 30 mg/kg MT group and n = 5 in other group. (**D**) n = 5 in MT 3 mg/kg and 15 mg/kg group, n = 6 in other group. *P ≤ 0.05, **P ≤ 0.01, ***P ≤ 0.001, N.S., not significant. Tukey’s post-hoc analysis. (**E**) The time processing curves for the scratching behaviors of the animals treated with AEW (n = 18) and water (n = 5). The symptom of chronic itching was basically stable after the five days. *P ≤ 0.05, **P ≤ 0.01, ***P ≤ 0.001, unpaired student’s *t*-test. (**F**) 30 mg/kg MT (s.c.) (n = 7) significantly decreased the scratching behaviors in the AEW chronic pruritus model on the 7^th^ day compared to saline (n = 5) group. ***P ≤ 0.001, N.S., not significant, Tukey’s post-hoc analysis. (**G**) The time processing curves for the scratching behaviors of the animals treated with methods of AD model (n = 16) and olive oil (n = 8). The symptom of chronic itching was basically stable after nine days. **P ≤ 0.01, ***P ≤ 0.001, unpaired student’s *t*-test. (**H**) 30 mg/kg MT (s.c.) (n = 6) significantly decreased the scratching behaviors in the AD chronic pruritus model compared saline group (n = 5) on the 12^th^ day. ***P ≤ 0.001, N.S., not significant, Tukey’s post-hoc analysis.

*Sophorae Flavescentis Radix (SFR)* has been used for treatment of viral hepatitis, cancer, enteritis, viral myocarditis, arrhythmia, and skin diseases in China, Japan and some European countries^22–24^. MT is a tetracycloquinolizindine alkaloid from *SFR* and is also its major component (chemical structure is shown in Fig. 1A)^25,26^. MT exhibits many biological activities and possesses a wide range of pharmacological effects. MT is associated with antinociceptive effect on mechanical and cold stimuli in a mouse model of vincristine-induced neuropathic pain; the antinociceptive effect of MT is mediated mainly through activation of к opioid receptors and partially through µ opioid receptors^27^. Because the skeleton structure of the MT is completely different from those of conventional к opioid receptors agonists, such as ethylketocyclazocine and nalfurafine, the framework of MT has been a candidate for screening new analgesic drug that might not induce side effects such as dysphoria and psychotomimetic actions. A lead compound was identified by determining the essential structure required for the antinociceptive effects of MT^28,29^. Derivatives of this compound were synthesized with a variety of phenyl substituents and evaluated for their antinociceptive effects^30^.

Compared with these advances, studies on the anti-pruritus of *SFR* are relatively few. Because *SFR* has specific therapeutic effects on pruritus and allergic symptoms with less side effects, it has become a very important candidate for some itch researchers to study on antipruritic drugs. *SFR* might alleviate allergic symptoms by inhibition of histamine signaling including mRNA levels of histamine H1 receptor (H1R) and histidine decarboxylase (HDC), H1R and HDC activities and histamine content^31,32^. When administrated orally at a dose of 200 mg/kg, *SFR* significantly inhibited serotonin (5-HT)-induced itch behaviors and spontaneous scratching bouts of mice induced by an atopic dermatitis (AD) model; the inhibitory effect of *SFR* on 5-HT was a dose-dependent manner^33^.

These studies suggested that *SFR* and MT inhibited not only the histamine-dependent but also the histamine-independent-induced itch; however, the type and mechanism of *SFR* and MT anti-pruritus remained unclear. Therefore, in the present study, the antipruritic effects and underlying mechanisms of MT were investigated at the behavioral, cellular and molecular levels. We demonstrated that MT could obviously inhibit different types of itching, including acute and chronic pruritus. Furthermore, the antipruritic effect of MT was achieved by inhibiting the N-type calcium channel on DRG neurons and blocking the synaptic transmission of DRG to the dorsal horn in the spinal cord. Our findings suggest that MT could be developed into a novel drug candidate in treating pruritus disorders, especially for some patients with chronic pruritus that are difficult to deal with.

## Results

### MT could inhibit acute and chronic pruritus

MT was a pyridine alkaloid compound; its molecular formula was shown in Fig. 1A. Histamine, chloroquine and compound 48/80 were used to induce acute itch in mice. Four doses of MT (30 mg/kg, 15 mg/kg, 6 mg/kg and 3 mg/kg) were designed by experiments to detect their inhibitory roles. The effects of MT on acute pruritus by subcutaneous injection of histamine, chloroquine and compound 48/80 are shown in Fig. 1B–D. After 30 min of MT treatment, the scratching behaviors induced by histamine (200 mM) were significantly decreased in a MT dose-dependent manner. The number of scratches reduced from 167.80 ± 13.56 (control, n = 5) to 37.60 ± 8.99 (30 mg/kg MT, n = 5, *P* < 0.001), 113.80 ± 16.84 (15 mg/kg MT, n = 5, *P* < 0.05), respectively. However, when the dose of MT reduced to 6 mg/kg, it had no obvious inhibitory effect. If the dose of MT was further decreased to 3 mg/kg, the antipruritic effect disappeared. The number of scratches were 153.7 ± 18.63 (6 mg/kg MT, n = 6, *P* = 0.5697) and 168.0 ± 26.59 (3 mg/kg MT, n = 5, *P* = 0.9948), respectively (Fig. 1B).

MT could effectively inhibit the itching induced by chloroquine (CQ) (8 mM); the inhibitory effect of MT on chloroquine was stronger than that of histamine. The number of scratches in mice treated with different doses of MT reduced from 222.3 ± 10.64 (control, n = 7) to 6.857 ± 3.003 (30 mg/kg MT, n = 7, *P* < 0.001), 53.33 ± 21.54 (15 mg/kg MT, n = 5, *P* < 0.001), and 79.00 ± 14.05 (6 mg/kg MT, n = 5, *P* < 0.001), respectively. And 3 mg/kg MT (257.3 ± 12.02, n = 5, *P* = 0.9170) had no effect on the scratching behaviors compared to control (Fig. 1C).

Compound 48/80 induced itch production by promoting histamine release and mast cell degranulation. The inhibitory effect of MT on compound 48/80-induced itching was similar to that of histamine. Compound 48/80-induced scratching numbers under different doses of MT were reduced from 308.2 ± 18.58 (control, n = 6) to 25.83 ± 10.06 (30 mg/kg MT, n = 6, *P* < 0.001), 174.6 ± 22.36 (15 mg/kg MT, n = 5, *P* < 0.01), respectively. 6 mg/kg MT (274.5 ± 22.97, n = 6, *P* = 0.2811) and 3 mg/kg MT (323.0 ± 21.84, n = 5, *P* = 0.6211) had no effect on the scratching behaviors compared to control (Fig. 1D).

AEW and AD models were employed in the MT test for chronic itching suppression. After two days of AEW treatment, the animals appeared to have obvious symptoms of scratching. With the prolonging of treatment time, the animals’ scratching behaviors further increased. Five days later, the number of scratches reached a maximum and in the next few days remained stable (Fig. 1E). Scratching behaviors on the 7^th^ day in the AEW model could obviously be inhibited by 30 mg/kg MT (s.c.). The number of scratches significantly reduced from 100.4 ± 12.49 (saline, n = 5) to 11.57 ± 8.15 (30 mg/kg MT, n = 7, *P* < 0.001). The control group (n = 6) compared with 30 mg/kg MT group (n = 7) were 83.83 ± 18.55 and 11.57 ± 8.15 (*P* < 0.01). However, the control group (n = 6) and the saline group (n = 5) were 83.83 ± 18.55 and 100.4 ± 12.49, with no significant difference (Fig. 1F).

A similar result also appeared on the AD model; the animal showed the pruritus syndrome in the process of AD modeling (Fig. 1G). Nine days later, the scratching behaviors reached the maximum and maintained a stable state. Then, MT was used for the detection of itch behaviors in the AD model; 30 mg/kg MT (s.c.) could significantly inhibit the pruritus behaviors of the AD model mice compared to the saline group on the 12^th^ day (238.2 ± 42.24, n = 5 *vs*. 10.17 ± 6.95, n = 6, *P* < 0.001). The control group (n = 5) compared with 30 mg/kg MT group (n = 6) on the 12^th^ day were 264.4 ± 41.39 and 10.17 ± 6.95 (*P* < 0.001). The control group (n = 5) and the saline group (n = 5) were 264.4 ± 41.39 and 238.2 ± 42.24 (*P* = 0.6695) (Fig. 1H). All results suggested that MT had an inhibition effect on chronic pruritus.

### MT neither inhibited the activity nor affected the survival of DRG neurons

**Figure 2:**
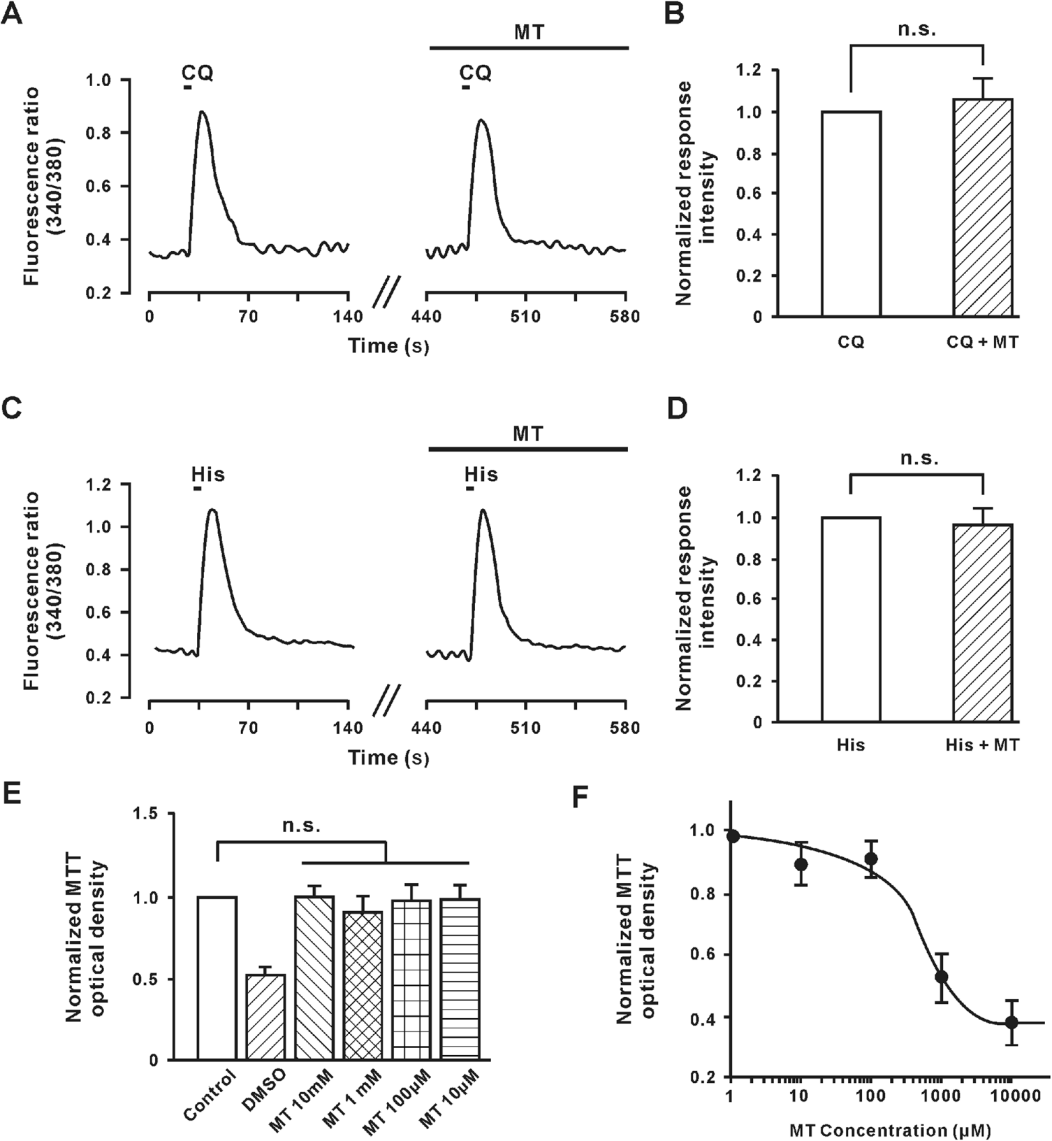
MT did not influence the Ca^2+^ influx evoked by chloroquine and histamine and survival of DRG neurons. (**A**) The representative trace displayed intracellular Ca^2+^ influx induced by 1 mM chloroquine, and 100 µM MT showed no effect to DRG neurons. MT did not influence the baseline too. (**B**) MT did not change the intensity of chloroquine-induced calcium influx in DRG neurons, n = 25 in each group. N.S., not significant, paired student’s *t*-test. (**C**) The representative trace displayed intracellular calcium flux induced by 1 mM histamine, and 100 µM MT showed no effect on DRG neurons. (**D**) MT did not change the intensity of histamine-induced calcium influx in DRG neurons, n = 40 in each group. N.S., not significant, paired student’s *t*-test. (**E**) The MTT optical density of alive DRG neurons before (n = 5) and after being treated by pure DMSO (n = 3), 10 mM MT (n = 4), 1 mM MT (n = 4), 100 μM MT (n = 4) and 10 μM MT (n = 4). N.S., not significant. Tukey’s post-hoc analysis. (**F**) The dose-response curve of MT to inhibit the MTT optical density of alive HEK293 cells. n = 5 in each concentration. Group data presented by mean ± SEM.

MT has been able to suppress acute and chronic pruritus. DRG is an important class of neurons that receive peripheral sensation including itch signal and send it to the spinal cord. We would like to detect whether the MT inhibited the activity of DRG, so we applied calcium imaging, and conducted cytotoxicity experiments. As shown in Fig. 2A–D, chloroquine (1 mM) and histamine (1 mM) evoked a remarkable Ca^2+^ influx in the DRG neurons. However, by pre-perfusion with 100 μM MT of the same neurons, the intensity of chloroquine and histamine-induced response did not change. The changes of reaction intensity were from 1 to 1.06 ± 0.092 (n = 25, *P* = 0.4598) in chloroquine (Fig. 2A,B) and from 1 to 0.9586 ± 0.0841 (n = 40, *P* = 0.6255) in histamine (Fig. 2C,D).

Furthermore, we explored the 3-(4,5)-dimethylthiahiazol(-z-y1)-3,5-di-phenytetrazoliumromide (MTT) assay to test the toxicity of the different MT concentrations in culture DRG neurons. As shown in Fig. 2E, MT did not affect the survival of DRG neurons, even at the concentration that reached 10 mM. However, in contrast to DRG neurons, the growth of HEK293 cells could be inhibited by MT and had dose-dependent properties (Fig. 2F). Although the IC50 of MT on growth of HEK293 cells was 141.37 μM, there was no statistically significant MTT optical density between 100 μM MT and the control group. Together, these results suggest that MT (100 μM) neither inhibited the activity nor affected the survival of DRG neurons.

### Intraperitoneal injection of MT had the same anti-pruritus effect

**Figure 3:**
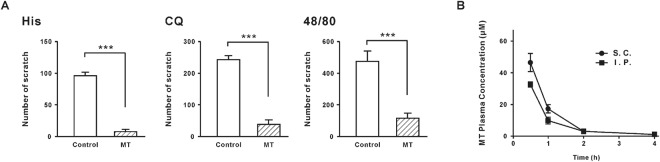
Antipruritic effect and detection in blood of MT intraperitoneal injection. (**A**) Like subcutaneous injection, intraperitoneal injection of 30 mg/kg MT also significantly decreased the scratching behavior induced by 200 mM histamine, 8 mM chloroquine and 13 mM compound 48/80, n = 4 in each group. ***P ≤ 0.001, unpaired student’s *t*-test. (**B**) Average plasma concentration-time relationships curves of MT after intraperitoneal or subcutaneous injection of 30 mg/kg MT, n = 3 in each time point. Group data presented by mean ± SEM.

Intraperitoneal injection of MT had a similar inhibitory effect on itching. Intraperitoneal injection of MT 30 mg/kg could significantly decrease the scratching behaviors induced by histamine (200 mM), chloroquine (8 mM) and compound 48/80 (13 mM). The scratching numbers reduced from 96.00 ± 6.11 to 7.50 ± 4.03 (n = 3, *P* < 0.001) (histamine), from 244.01 ± 11.55 to 38.00 ± 12.59 (n = 4, *P* < 0.001) (chloroquine) and from 475.23 ± 64.86 to 118.30 ± 33.10 (n = 4, *P* < 0.001) (compound 48/80) (Fig. 3A).

These results indicate that MT could act not only at the periphery, but also as a function of the center. Since MT is a compound that is easily soluble in water, it will quickly be able to get into the bloodstream. To make sure of the time MT was stuck in the blood, we measured the changes in blood concentrations of MT after 0.5, 1, 2, and 4 hours. As shown in Fig. 3B, the concentrations in the blood after subcutaneous injection of 30 mg/kg MT were 46.56 ± 5.56 µM (n = 3), 17.22 ± 2.57 µM (n = 3), 3.21 ± 0.46 µM (n = 3) and 0.33 ± 0.03 µM (n = 3) at 0.5, 1, 2, and 4 hours. As expected, intraperitoneal injection of MT 30 mg/kg could also enter the blood circulation, and the concentration-time curve was consistent with the subcutaneous injection. The blood concentrations of MT were 32.79 ± 1.41 µM (n = 3), 9.99 ± 1.69 µM (n = 3), 2.94 ± 0.55 µM (n = 3) and 0.23 ± 0.03 µM (n = 3) at 0.5, 1, 2, and 4 hours. The same effect of MT on relieving itching by neck subcutaneous injection and intraperitoneal injection indicated that MT might play a role in spinal cord segments through the circulation of blood.

### MT mainly inhibited N-type calcium channel on DRG neurons

MT neither affected the survival of DRG cells nor changed the Ca^2+^ influx of chloroquine and histamine-induced responses. Next, we asked whether the inhibition of MT altered the function of certain ion channels on DRG neurons. We examined different ion channels on DRG neurons and found that MT inhibited calcium-related ion channels.

**Figure 4:**
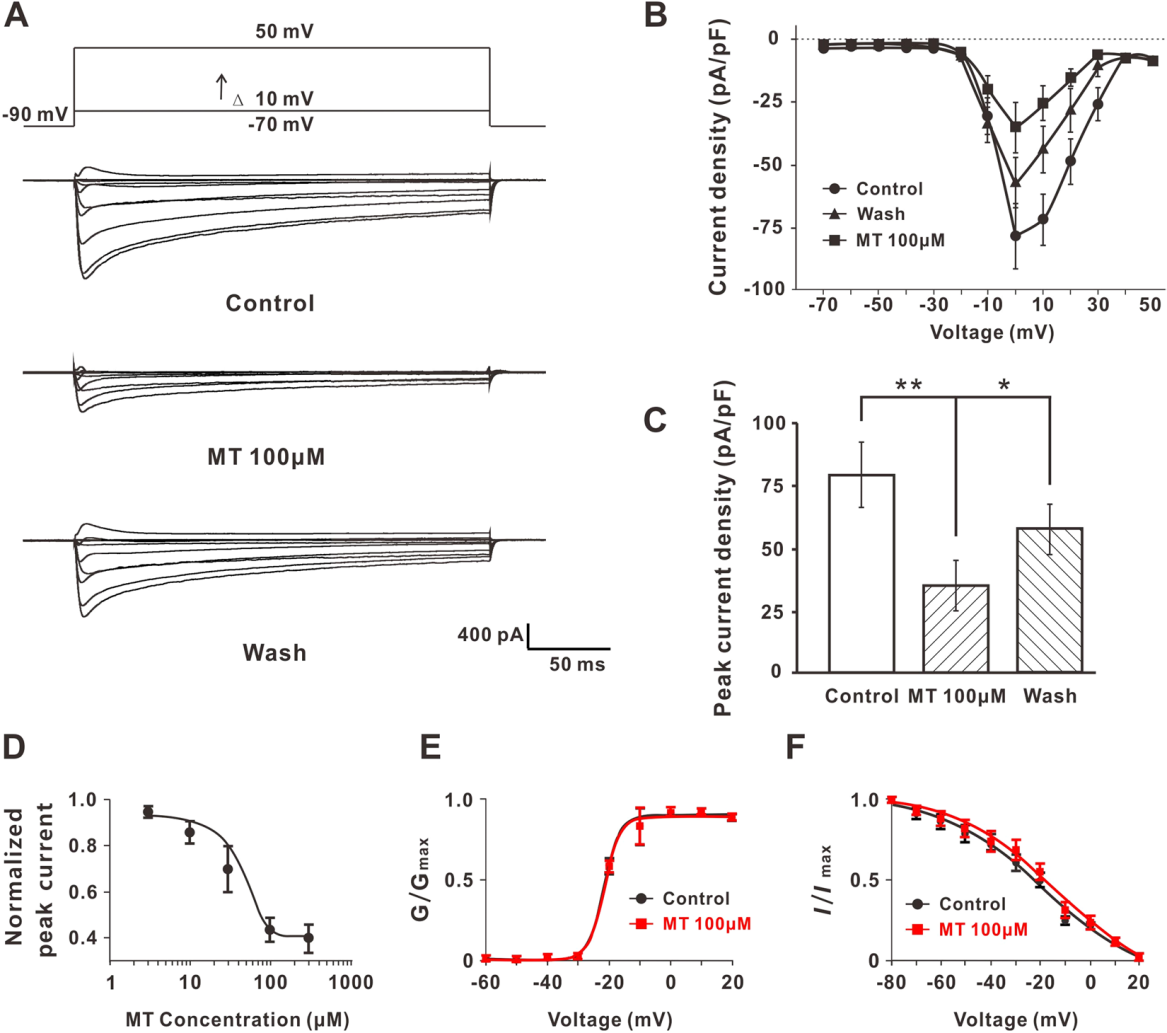
MT inhibited Ca^2+^ currents in DRG neurons. (**A**) MT attenuated the whole cell Ca^2+^ currents evoked by a step depolarization in DRG neurons and the effect could be washed out. (**B**) Average whole cell Ca^2+^ current density-voltage relationships curves of tested DRG neurons under 100 µM MT perfusion and washed out (n = 5). (**C**) The peak current density in MT perfusion and washout states. n = 5 in each group. *P ≤ 0.05, **P ≤ 0.01. Tukey’s post-hoc analysis. (**D**) Dose-response curve of MT for peak Ca^2+^ current, n = 5 in each point. (**E**) Activation curve of whole cell calcium channels before and after 100 µM MT perfusion, n = 5 in each group. (**F**) Inactivation curve of whole cell calcium channels before and after 100 µM MT perfusion, n = 5 in each group. Group data presented by mean ± SEM.

As shown in Fig. 4A, the total Ca^2+^ currents were evoked by a train of depolarizing steps command voltage (Δ = 10 mV) from −70 to +50 mV with a duration of 300 ms. After 100 µM MT perfusion, the total Ca^2+^ currents significantly decreased (Fig. 4A,B). The peak current density of total Ca^2+^ currents were −80.23 ± −13.22 vs. −32.56 ± −9.96 pA/pF (n = 5, *P* < 0.01) (Fig. 4C) before and after MT perfusion. MT-induced inhibition diminished after washout with extracellular solution, which suggested that the drug action was reversible. Next, we infused the DRG neurons with different concentrations of MT (3, 10, 30, 100 and 300 µM) and made the dose-Ca^2+^ currents curve (Fig. 4D). The half-maximal inhibitory concentration (IC50) of MT was 42.01 µM. We found that although we raised the concentration to 1 mM, the inhibitory effect of MT did not change significantly compared to 300 µM MT. It suggested that the MT Cmax was 300 µM, and that the 100 µM MT that we used was a suitable concentration. We also tested the effect of MT on the activation and inactivation of Ca^2+^ channels; however, the activation and inactivation curve of Ca^2+^ channels had no significant difference before and after 100 µM MT perfusion. The *V*_1/2 act_ and *V*_1/2 inact_ were −21.57 mV vs. −21.60 mV and −19.01 mV vs. −17.8 mV before and after 100 µM MT perfusion, respectively (Fig. 4E,F).

**Figure 5:**
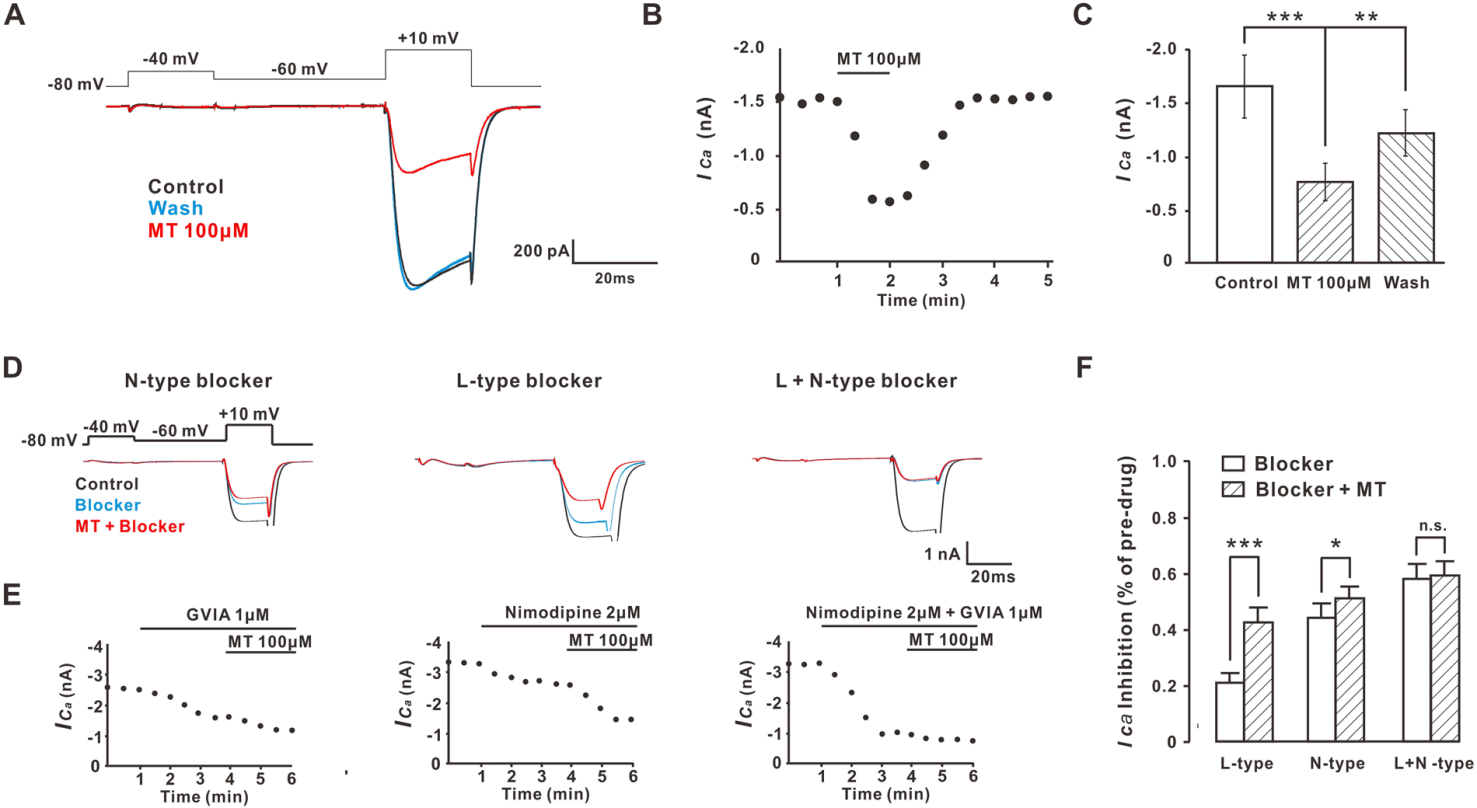
MT inhibited HVA Ca^2+^ currents of N-type and L-type calcium channel. (**A**) The protocol of stimulus and traces of LVA *I*_*Ca*_ (−80 to −40 mV, 20 ms) and HVA *I*_*Ca*_ (−60 to 10 mV, 20 ms). Inward current traces were evoked by depolarization during bath application of MT and washed out. (**B**) Current-time curves of MT to inhibit the HVA *I*_*Ca*_. (**C**) Histogram of HVA *I*_*Ca*_ current intensity during application and washout of MT, n = 10 in each group. **P ≤ 0.01, ***P ≤ 0.001, Tukey’s post-hoc analysis. (**D**) Stimulation protocols for inducing calcium channels and current traces under blockers of N-type (GVIA, 1 µM), L-type (nimodipine, 2 µM) and N-type + L-type. On the same neurons to run the protocol, the inhibitory effect of 100 µM MT (red line) was also detected and compared with the inhibitory effect of other calcium channels blockers. (**E**) The current-time curve relationships of three substances (MT, GVIA and nimodipine) to inhibit HVA *I*_*Ca*_. (**F**) The inhibition percentage of N-type blockers (n = 12), L-type blockers (n = 11) and N- and L-type blockers together (n = 14) with and without MT perfusion to inhibit HVA *I*_ca_ current. MT could inhibit the action of N-type and L-type calcium channels, but the inhibitory effect of MT on N-type was stronger than that of L-type. *P ≤ 0.05, ***P ≤ 0.001, N.S., not significant, paired student’s *t*-test. Group data presented by mean ± SEM.

Since the voltage-gated Ca^2+^ channels were divided into two categories, low-voltage-activated (LVA) and high-voltage-activated (HVA), we wanted to know which type of calcium channels the MT worked on. The results indicated that the application of 100 µM MT in bath solution strongly inhibited HVA *I*_*ca*_ but not LVA *I*_*ca*_ (Fig. 5A). Current-time curves also showed that the HVA *I*_ca_ current was decreased with MT addition; after MT perfusion, it recovered gradually (Fig. 5B). The HVA *I*_ca_ reduced from 1.644 ± 0.362 to 0.743 ± 0.216 nA (n = 10, *P* < 0.001) (Fig. 5C). Then, we determined which subtype of HVA channel was modulated by MT. We treated neurons with antagonist to N-type (ω-conotoxin GVIA, 1 µM, n = 11) or L-type (nimodipine, 2 µM, n = 12) channel, and then added MT to the same neuron. We found that N-type or L-type blockers alone could partly block the inhibition effect of MT on HVA *I*_ca_ current; however, the two blockers together totally blocked the effect of MT (Fig. 5D–F). These findings suggested that MT inhibited both N-type and L-type channels in mouse DRG neurons. In addition, compared to N-type blocker alone, the inhibition effect of N-type blocker and MT was not very significant: the inhibitory rates were 44.25 ± 5.09% and 50.90 ± 5.09% (n = 12, *P* < 0.05), respectively. By contrast, the inhibitory rates of L-type blocker alone and MT were 21.10 ± 3.92% and 42.76 ± 6.08% (n = 11, *P* < 0.001); there were very significant statistical differences (Fig. 5F). These results suggest that the inhibitory effect of MT on HVA *I*_ca_ current is mainly by inhibiting the N-type calcium channel.

### The inhibitory effect of MT was on the presynaptic membrane rather than on the postsynaptic membrane in lamina II neurons

N-type calcium channel played a vital role in excitatory synaptic transmission, and MT mainly inhibited the N-type calcium channel in mouse DRG neurons. We naturally thought that MT might be involved in the transmission of peripheral signals to the center. To obtain data support, we completed electrophysiological experiments on superficial neurons in the dorsal horn of the spinal cord slices.

**Figure 6:**
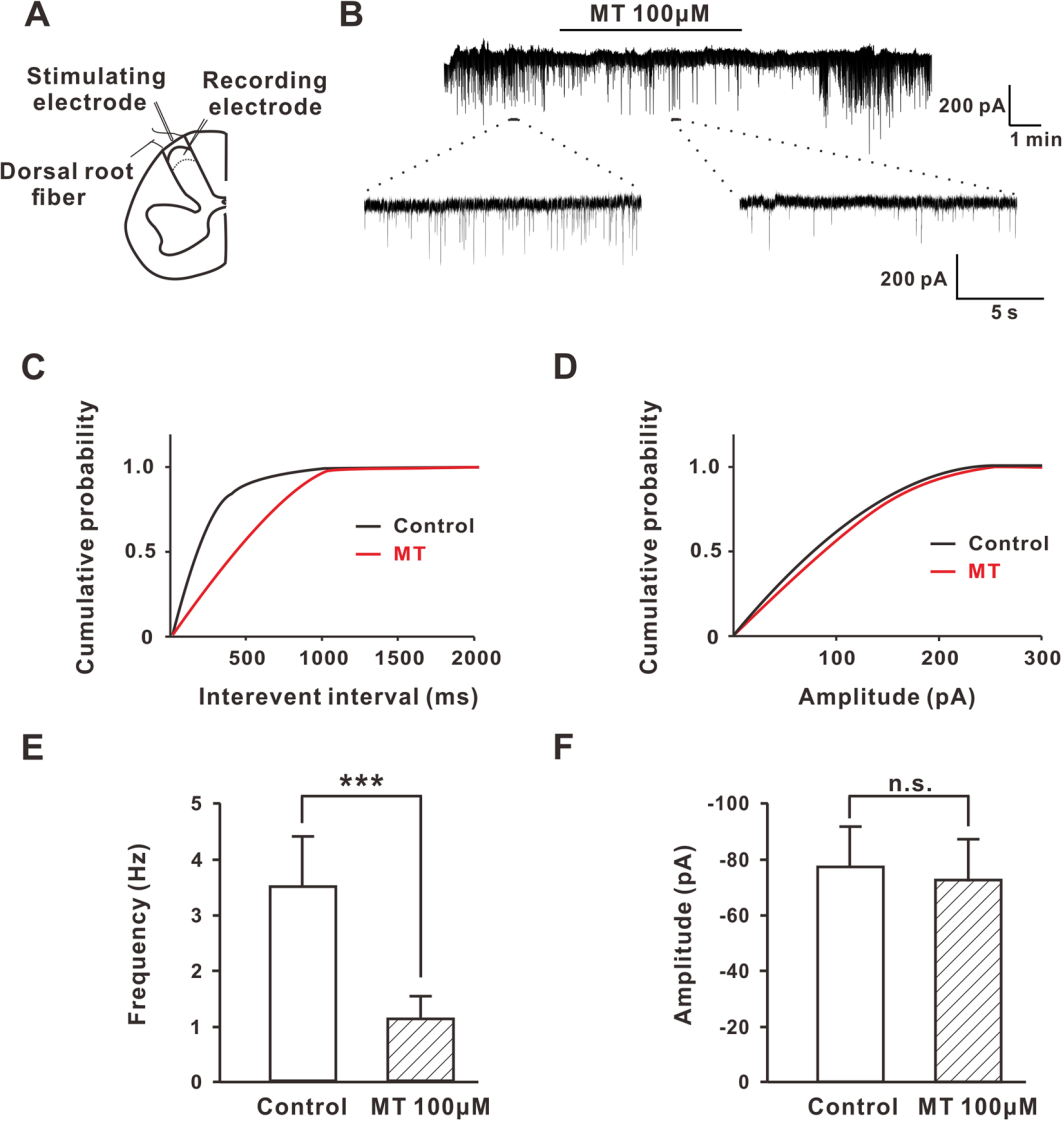
MT inhibited the frequency instead of amplitude of glutamatergic sEPSCs in lamina II neurons. (**A**) Patterns of electrode recording and stimulation sites were shown on spinal cord slice. The recording electrode was located on the *substantia gelatinosa* (SG, lamina II) neuron, and the stimulating electrode was located in the dorsal root of the spinal cord. (**B**) Spontaneous inward currents were recorded and the inhibitory effect of 100 μM MT on the *substantia gelatinosa* (SG, lamina II) neurons was shown. (**C**,**D**) Cumulative plots showed changes in the frequency and amplitude of glutamatergic sEPSCs after 100 μM MT perfusion in lamina II neurons. (**E**,**F**) The perfusion of MT reduced the firing frequency (n = 7) of the lamina II neurons, but did not change the amplitude (n = 5) of sEPSCs. ***P ≤ 0.001, N.S., not significant, paired student’s *t*-test. Group data presented by mean ± SEM.

In lamina II of the spinal cord slices, the baseline frequency of spontaneous excitatory postsynaptic current (sEPSCs) was 3.51 ± 0.87 Hz in seven recorded neurons. When using 100 µM MT to incubate the spinal cord slices, the frequency of sEPSCs obviously decreased from 3.51 ± 0.87 Hz to 1.01 ± 0.42 Hz (n = 5, *P* < 0.001) (Fig. 6B,C and E), but the amplitudes of sEPSCs in these neurons had no significant effects and were −77.83 ± 14.51 mV and −71.53 ± 15.62 mV (n = 5, *P* = 0.316, paired *t*-test) (Fig. 6B,D and F). These results suggest that MT inhibits the presynaptic release of glutamate rather than postsynaptic glutamate action.

**Figure 7:**
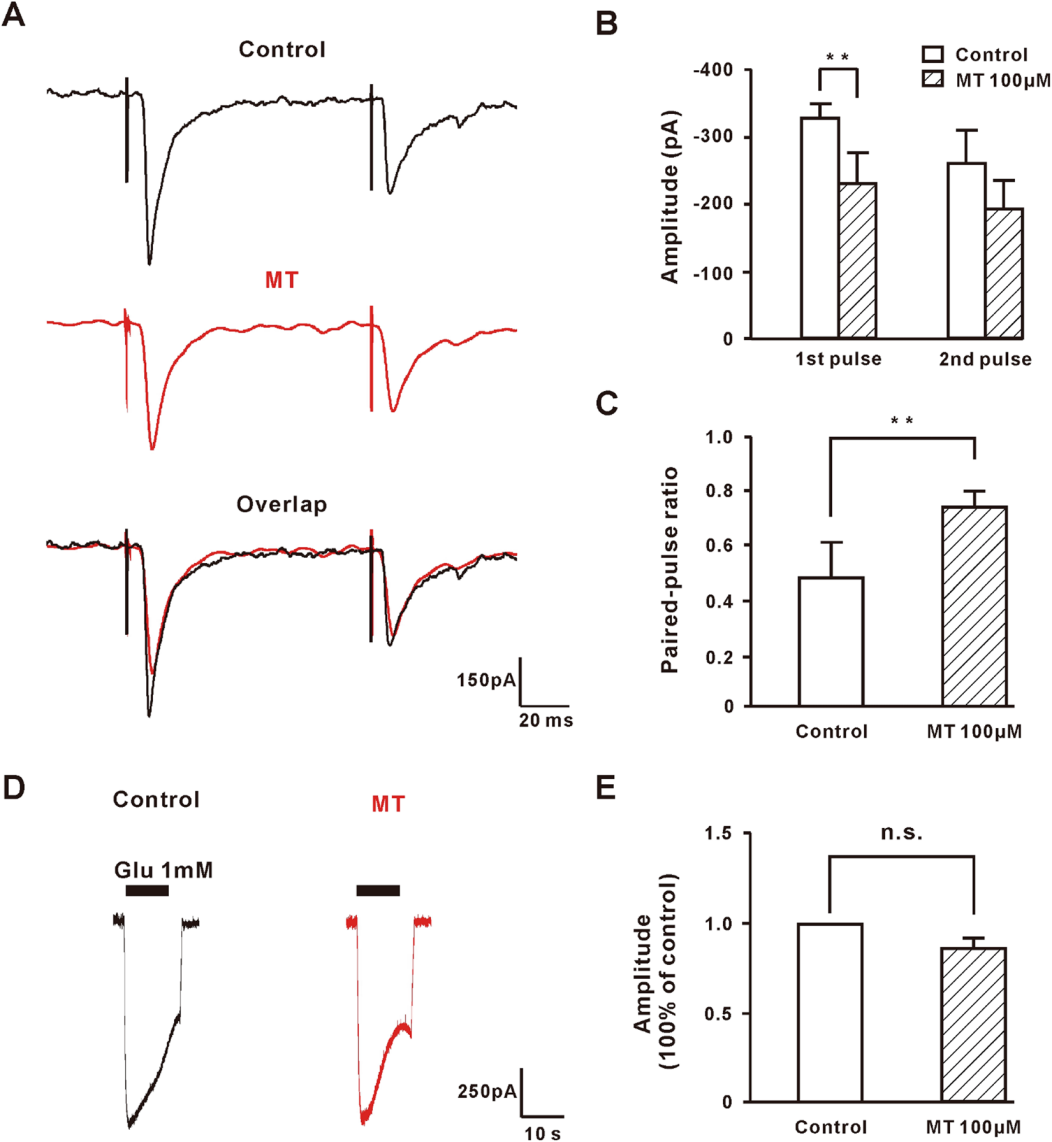
MT inhibited the evoked synaptic responses in lamina II neurons, but the inward currents induced by exogenous glutamic acid were not affected. (**A**) The response traces of evoked excitatory postsynaptic currents (eEPSCs) to high-intensity, paired-pulse stimulation (500 µA, 0.1 ms, 400 ms interval) and application of 100 µM MT to lamina II neurons. The black and red lines indicated the response of the control and the 100 µM MT administration. (**B**) MT obviously decreased the eEPSC amplitude (n = 6). **P ≤ 0.01, paired student’s *t*-test. (**C**) MT significantly increased the paired-pulse ratio (PPR; 2^nd^ eEPSC amplitude/1^st^ eEPSC amplitude) (n = 6). **P ≤ 0.01, paired student’s *t*-test. (**D**) Whole-cell currents evoked by exogenous application of glutamate and 100 µM MT perfusion. (**E**) MT did not affect exogenous glutamate induced inward currents (n = 5). N.S., not significant, paired student’s *t*-test. Group data presented by mean ± SEM.

To elucidate the presynaptic inhibitory effect of MT on the spinal dorsal horn, we recorded evoked excitatory postsynaptic current (eEPSCs) after high-intensity paired-pulse stimulation (150–500 µA, 0.1 ms, 400 ms apart, 3 tests/min) applied at the dorsal root. We used paired-pulse stimulation to calculate the paired-pulse ratio (PPR; 2^nd^ eEPSC amplitude/1^st^ eEPSC amplitude). The change in PPR was used to assess whether the inhibition of postsynaptic response by a drug might involve a decrease in presynaptic release of excitatory neurotransmitters. We observed that there was a statistically significant decrease of first eEPSC amplitude after MT perfusion (Fig. 7A,B). Also, the application of MT significantly increased the paired-pulse ratio from 0.49 ± 0.13 to 0.77 ± 0.06 (n = 6, *P* < 0.01) in responsive cells (Fig. 7C), in accordance with the results of sEPSC that MT inhibited the presynaptic release of glutamate. Moreover, to eliminate the postsynaptic effect of MT on glutamatergic EPSC, we tested whether MT would change current responses elicited by exogenous application of glutamate (1 mM). MT caused no change (n = 5, 86.56 ± 0.06% of control, *P* = 0.067) in amplitude of inward current induced by glutamate (Fig. 7D,E). This also suggests that the action of MT is a presynaptic rather than a postsynaptic mechanism.

## Discussion

Pruritus is a very uncomfortable feeling that leads to the desire or reflex to scratch. Approximately one-tenth of the population suffers from this symptom, and this rate is even higher among old people^1^. It seriously affects the quality of life of patients. However, causes of pruritus are complex, which makes it difficult to cure. In the clinic, anti-histamines are the most commonly used antipruritic drugs, but the scope of treatment is very limited. Until now, the vast majority of histamine-independent and chronic pruritus has had no effective drug or therapy treatments, and it becomes a worldwide problem^34,35^. MT has already been used to treat some diseases such as hepatitis, enteritis, and atopic dermatitis in clinics in China^36^. Because the medicinal qualities of anti-inflammation and immunity-regulation, intramuscular or intravenous injection of MT can also alleviate and cure chronic HBV, eczema and contact dermatitis, which are usually accompanied by itching symptoms^37,38^.

In the present study, we described a role for MT in antipruritic effects. First, subcutaneous injection of MT could inhibit the scratching behaviors not only induced by histamine, chloroquine and compound 48/80 with a dose-dependent manner, but also in chronic pruritus models of AD and AEW. Second, intraperitoneal injection of MT also had the same antipruritic effect as subcutaneous injection of MT. MT could be detected in the blood after the two injection methods. This suggested that MT might get into the spinal cord via blood circulation. Finally, electrophysiological experiments demonstrated that MT inhibited the excitatory synaptic transmission from DRG to the dorsal horn of the spinal cord by suppressing presynaptic N-type calcium channel, instead of by direct inhibition of DRG neuronal activity. These results provided strong evidence that MT had great potential value in the treatment of pruritus.

In consideration of so many kinds of pruritus that MT could inhibit, we speculated that the mechanism of the antipruritic effect of MT might be involved in the inhibition of production or transmission of itch signals. However, our calcium imaging results showed that MT itself did not directly act on the DRG neurons. Moreover, MT did not influence histamine or chloroquine-induced calcium influx in DRG neurons. These results suggested that MT could not directly inhibit the ligand-induced calcium influx of soma of DRG neurons to relieve itching.

Voltage-gated calcium channels (VGCC) are expressed in most excitable cells, playing an important role in neuronal excitability and neurotransmitter release^39–42^. It is well recognized that all VGCC subtypes are found expressed throughout the DRG and the spinal cord, with a prominent expression of N-type VGCC, which are essential for cellular signaling and are key participants in peripheral sensitization^43,44^. Inhibition of N-type channels, such as N-type antagonist MVIIA and Phα1β, is a mechanism for inhibiting neurotransmitter release and spinal itch signal transmission^45^. Our findings showed that MT inhibited the HVA *Ica*, and that the inhibition of MT could be prevented by pretreatment with a blocker of N-type. The influence of MT on the N-type channels demonstrated that MT might attenuate excitatory neurotransmission in the superficial dorsal horn cells that received input from the DRG. Accordingly, bath application of MT to spinal cord slices decreased the frequency instead of the amplitude of sEPSC. Furthermore, MT also inhibited the eEPSC and increased the PPR in these superficial dorsal horn cells, which suggested that it might involve a decrease in excitatory neurotransmitter release from central terminals of DRG neurons. Also, MT did not block the inward current induced by glutamic acid that was exogenously added. All together, these findings pointed to a presynaptic instead of postsynaptic mechanism of MT to the inhibition of neurotransmitter release and spinal itch transmission.

It is well known that N-type channel in DRG neurons is important for cellular signaling and is a key participant in peripheral sensitization that leads to exaggerated pain^46,47^. It is not difficult to think of the inhibition of MT on the N-type channels of DRG suggesting that MT may have an analgesic effect. Indeed, in both chronic constriction injury and chemically induced neuropathic pain models, MT at the dose of 30 mg/kg (i.p.) increased the paw withdrawal threshold, paw withdrawal latency, and the counts of paw withdrawal compared to the control groups^48,49^. In addition, MT attenuated the expression of inflammatory and oxidative stress factors in these neuropathic pain models^48^. Our results showed that MT could achieve significant antipruritic effect by inhibiting N-type channel, after half an hour intraperitoneal or subcutaneous injection of MT. It seemed that the analgesic effect and antipruritic effect of MT were not accomplished by the same mechanism. Previous studies found that pretreatment with a kappa-opioid receptor (KOR) antagonist attenuated but did not completely block the MT-induced antinociceptive effect in the mouse tail-flick and warm-plate test^27,50,51^. However, another report showed that the antinociceptive effect of MT was attenuated by muscarinic receptor antagonist atropine and pirenzepine but not by naloxone, which was a peripherally acting opioid receptor. And MT had no affinity for mu-, kappa- or delta-opioid receptors^52^. These results suggested that even KOR could block the effect of MT, but there were still other mechanisms for the analgesic effect. Actually, neuropathic pain has been linked with a rise in neuronal calcium levels followed by enhancement of the oxidative stress markers (free radicals) and inflammatory cytokines, etc.^53,54^. This suggested that the inhibitory effect of MT on neuropathic pain might also be mediated by the calcium channels.

The N-type calcium channel antagonist, MVIIA, was approved to be used clinically as a spinally delivered analgesic to control untreatable pain; it was also found to have a significant antipruritic effect^45^. But drugs such as MVIIA only work when they are administered by intrathecal injection. Compared to subcutaneous injection, intrathecal injection is less convenient and increases the risk of infection, internal bleeding, and spinal cord injury^55,56^. Our results showed that MT could be absorbed into the blood circulation by intraperitoneal and subcutaneous injection and sent into the spinal cord. A more convenient route of administration suggested that MT had more advantages as an antipruritic drug. In addition, 30 mg/kg MT of intraperitoneal injection did not alter the rotarod performance time or spontaneous locomotor activity compared to the control group^48,49^. Therefore, MT did not have an apparent effect on motor function. Moreover, the results of the MTT test indicated that even high concentrations of MT (up to 10 mM) had no influence on the survival of neurons. Considering the extensive clinical applications of MT in various diseases such as hepatitis^57^, all these results suggested that MT could be a powerful potential anti-pruritus drug without obvious side effects.

Taken together, to our knowledge, we demonstrated for the first time that inhibition of presynaptic N-type calcium channel was an important mechanism underlying the antipruritic effect of MT. Given the present results and the long history of safe application of *SFR* in anti-pruritus, we believe that our results provide a promising opportunity for developing new drugs in treating pruritus diseases, especially for histamine-independent and chronic pruritus.

## Experimental procedures

### Animals

C57BL/6 male mice (8–10 weeks) were used for behavioral testing (Experimental Animal Center, Nanjing University of Chinese Medicine, Nanjing, China). The study was performed in accordance with relevant guidelines and regulations of the Institutional Animal Care and Use Committee of the Nanjing University of Chinese Medicine. All experimental protocols were approved by the International Association for the Study of Pain.

### AEW and AD treatment

Mice were housed in a temperature-controlled animal room (22 ± 2 °C) under a 12-h light/dark cycle, with free access to food and water. They were acclimated to the testing environment for 30 min before the initiation of behavioral tests. And the animal behaviors were analyzed by investigators who were blind to genotype and animal treatment condition.

To experimentally induce dry skin, we treated the nape of the neck of mice with acetone-ether-water (AEW), as reported previously^58^. Animals were shaved at the nape of the neck in the first three days before starting treatment. A mixture of acetone and diethylether (1:1) was applied to the shaved area for 15 s, followed immediately by distilled water for 30 s. The control group just used cotton wetted by water for 45 s instead. The animals were treated twice daily.

For AD model, animals were shaved at the nape of the neck and abdomen fur, 150 μL 0.5% DNFB dissolved in an acetone: olive oil mixture (4:1) was applied into the abdomen for sensitization (day −4). From day 0 to day 11, 0.2% DNFB dissolved in an acetone: olive oil mixture (4:1) (model group) and olive oil (control group) were applied to challenge the shaved area of neck (50 μL) and three times a week.

### Behavioral test

For the chronic itch test, scratching behaviors in model mice with AEW or AD were surveyed for 30 min after molding treatment. On the 7^th^ day or 12^th^ day of treatment, we adopted subcutaneous injection of MT (30 mg/kg, 50 μL, dissolved in saline) or saline at the treated area. Then the scratching behaviors were observed for another 30 min. A bout of scratching was defined as a continuous scratching movement with a hind paw directed at the treated site or drug injection site. The bouts were obtained from the number of subtractions after injection.

For an acute experimental test, different concentrations of MT (3, 6, 15, 30 mg/kg) were treated with the nape of the neck by subcutaneous injection or intraperitoneal injection for 30 min. Then, the histamine (200 mM), chloroquine (8 mM), or compound 48/80 (13 mM) was injected into the same area of the neck and the scratching behaviors were observed for another 30 min.

### Culture of dissociated DRG neurons

DRG neurons were collected in ice cold DH10 medium (90% DMEM/F-12, 10% FBS, 100 U/mL penicillin, 100 mg/mL streptomycin, Gibco). Dissociated DRG neurons were then digested for 30 min at 37 °C in a protease solution (5 mg/mL dispase, 1 mg/mL collagenase type I in HBSS without Ca^2+^ or Mg^2+^, Gibco). After digestion, DRGs were triturated to free neurons and then neurons were collected by centrifugation (1000 rpm, 5 min). Cells were plated on the coverslips with poly-D-lysine (0.5 mg/mL, Sigma, Aldrich, USA) and laminin (10 mg/mL, Sigma, Aldrich, USA) coated, and 1‰ neuron growth factor (NGF, dissolved in DH10) was added into the media. All these cells were incubated at 37 °C, 5% CO_2_ for 16–18 h before the calcium imaging experiment.

### Whole-cell voltage-clamp recordings from DRG neurons

DRG neurons were identified by inverted microscopy (ZEISS, Axio Oberver D1, Germany). Coverslips were transferred into a chamber with the extracellular solution. Whole-cell current clamp and voltage-clamp recording experiments were performed at room temperature (23–25 °C) using a Multi-clamp 700B amplifier and Digital 1440 with pClamp10 software (Molecular Devices, USA).

Signals were sampled at 20 kHz and filtered at 2 kHz. The patch pipettes were pulled from borosilicate glass capillaries using a P-97 micropipette puller (Sutter Instrument) and had a resistance of 3–5 MΩ for patch-clamp recordings. The series resistance was routinely compensated at 60–80%. The resting membrane potential was recorded for each neuron under the current-clamp mode after stabilization (within 3 min). Neurons whose seal resistance were below 1 GΩ after breaking the cell membrane and whole-cell recording formation were excluded from analysis. The liquid junction potential was 8 mV and corrected. A single intact action potential was induced by a series of depolarizing current steps, each of 2 ms duration, increments of 50 pA through the recording electrode. The internal solution contained the following (in mM): KCl 135, MgATP 3, Na_2_ATP 0.5, CaCl_2_ 1, EGTA 2, glucose 5, with pH adjusted to 7.38 using KOH, and osmolarity adjusted to 300 mOsm with sucrose. The external solution contained the following (in mM): NaCl 140, KCl 4, CaCl_2_ 2, MgCl_2_ 2, HEPES 10, glucose 5, with pH adjusted to 7.4 using NaOH, and osmolarity adjusted to 310 mOsm with sucrose.

For voltage-gated calcium (Cav) current recording, the intracellular pipette solution contained (in mM): CsCl 135, CaCl_2_ 1, MgCl_2_ 2, MgATP 1.5, Na_2_GTP 0.3, EGTA 11, HEPES 10, with pH of 7.3 and osmolarity of 310 mOsm. The total calcium currents were evoked in response to depolarization steps to different testing potentials from −70 mV to +50 mV in 10 mV increments with a duration of 300 ms, preceded by a 500 ms prepulse of −90 mV. LVA *I*_ca_ was evoked at −40 mV (20 ms) from a holding potential of −80 mV, and HVA *I*_ca_ was evoked at 10 mV (20 ms) from a holding potential of −60 mV or −80 mV, repeated every 20 s. We used neurons with HVA *I*_ca_ (10 mV)/*I*_ca_ (−40 mV) > 1 for drug testing to limit the potential contamination of small HVA currents from residual LVA currents. The voltage dependence of current activation was obtained by depolarizing from −80 mV to +30 mV in 10 mV increments with a duration of 80 ms, preceded by a 100 ms prepulse of −70 mV, and estimated using a modified Boltzmann function to fit normalized I-V data: G/G_max_ = 1/(1 + exp ((*V*_1/2 act_ − V_m_)/κ)), where G is conductance, V_m_ is the test potential, *V*_1/2 act_ is the mid-point of activation, and κ is the slope factor. Steady-state inactivation relationships (or availability curves) were obtained by depolarizing to −10 mV for 50 ms after a prepulse of 500 ms depolarizing steps from −80 mV to +20 mV in 10 mV increments and estimated by fitting averaged data to a standard Boltzmann function: *I*/*I*_max_ = 1 + exp ((V_m_ − *V*_1/2 inact_)/κ), where *I*_max_ is the maximal current recorded at −30 mV, *V*_1/2 inact_ is the midpoint of steady-state inactivation, and κ is the slope.

### Whole-cell voltage-clamp recordings from spinal cord slices

To prepare spinal cord slices, we first performed a laminectomy in adult (4-week-old) C57BL/6 mice that were deeply anesthetized with CO_2_, then the lumbosacral segment of the spinal cord was rapidly removed and placed in ice-cold, low-sodium Krebs solution (in mM: NaCl 95, KCl 2.5, NaHCO_3_ 26, NaH_2_PO_4_-2H_2_O 1.25, MgCl_2_ 6, CaCl_2_ 1.5, glucose 25, sucrose 50, saturated with 95% O_2_/5% CO_2_). The tissue was trimmed and mounted on a tissue slicer (Vibratome VT1200, Leica Biosystems, Buffalo Grove, IL). Transverse slices (400 µm) with attached dorsal roots were prepared and incubated in normal Krebs solution (in mM: NaCl 125, KCl 2.5, NaHCO_3_ 26, NaH_2_PO_4_-2H_2_O 1.25, MgCl_2_ 1, CaCl_2_ 2, glucose 25, saturated with 95% O_2_/5% CO_2_). The slices recovered at 34 °C for 40 min and then at room temperature for an additional hour before being used for experimental recordings. For electrophysiology recording, slices were transferred to a low-volume recording chamber, which was perfused with normal Krebs solution at a rate of 2 mL/min and bubbled with a continuous flow of 95% O_2_/5% CO_2_. Whole-cell patch-clamp recording of lamina II cells was carried out under oblique illumination with an Olympus fixed-stage microscope system (FV1200, Olympus, Japan). Data were acquired by a Multi-clamp 700B amplifier and Digital 1550 with pClamp10 software (Molecular Devices, USA). We fabricated thin-walled glass pipettes (Sutter Instruments, Sarasota, FL) that had a resistance of 3–6 MΩ and were filled with internal solution (in mM): K-gluconate 120, KCl 20, MgCl_2_ 2, EGTA 0.5, Na_2_ATP 2, Na_2_GTP 0.5 and HEPES 20. To minimize inhibitory postsynaptic current contamination of EPSC recording, all recordings were made in the presence of SR95531 (10 μM) and strychnine (5 μM) in the external solution, to block GABA_A_ and Glycine receptor, respectively. A seal resistance of at least 1 GΩ and an access resistance of 20–35 MΩ were considered acceptable. Spontaneous EPSCs (sEPSCs) were recorded at a holding potential of −70 mV. For evoked EPSC (eEPSC), we delivered paired-pulse test stimulation to the dorsal root entry zone consisting of two synaptic volleys (150–500 µA, 0.1 ms) 400 ms apart at a frequency of 0.05 Hz. To study membrane currents elicited by exogenous transmitters, L-glutamate was applied from glass pipettes to the soma of the recorded neuron by short (10 s) pressure pulses.

### Calcium imaging

For calcium imaging experiments, the cells were loaded with Fura-2-acetomethoxyl ester (molecular Probes, Eugene, OR, USA) in HBSS solution for 30 min in the dark at room temperature. After washing 3 times, the glass coverslips were placed into a chamber and perfused with normal solution (in mM): NaCl 140, KCl 5, HEPES 10, CaCl_2_ 2, MgCl_2_ 2, glucose 10 and pH 7.4 with NaOH to adjust. A high-speed, continuously scanning, monochromatic light source (Polychrome V, Till Photonics, Gräfeling, Germany) was used for excitation at 340 and 380 nm, enabling us to detect changes in intracellular free calcium concentration.

### MTT assay

To investigate the effect of MT on the survival of cells, cell viability of DRG neurons or HEK293 cultured were tested by the MTT assay. Cells at a density of 5 × 10^3^ cells/well were seeded into each well in black well 96-well plates (Sigma, Aldrich, USA). After being cultured for 24 hours, cells were incubated by MT at 0.01, 0.1, 1 and 10 mM concentrations or pure DMSO. After another 24 hours, 10 µL tetrazolium bromide, 3-(4,5-dimethylthiazol-2-yl)-2,5-diphenyltetrazolium bromide (MTT, 1 mg/ml) (Sigma, Aldrich, USA) was added and cells were cultured at 37 °C for 4 hours. And then the medium was removed and DMSO (100 µL) was added. The optical density as the parameter of cell viability was measured at 570 nm with a microplate reader (Multiskan EX, Thermo, Ventana, Finland).

### HPLC-MS/MS assay

Twelve C57BL/6 mice were divided into two groups: One group of animals was shaved at the nape of the neck and the MT 30 mg/kg was injected by subcutaneous injection; the other group was injected MT 30 mg/kg with intraperitoneal injection manner. Blood samples were collected from mandibular vein at 0.5, 1, 2 and 4 hours after MT treatment. Then, the samples were centrifuged at 3000 rpm for 5 min and the plasma was isolated. The plasma samples (10 μL) were added to the acetonitrile solution (200 μL) containing 10 ng/mL dexamethasone and the mixture (1 μL) was prepared for HPLC-MS/MS test. The following modular HPLC-MS/MS system was used: Agilent 1200 HPLC-MS/MS instrument (Agilent, USA). The analytical column (2.1 × 150 mm, 2.5 µm) was packed with Kromasil C18 (2.5 µm). The mobile phase was composed of acetonitrile and water (50:50). The flow rate was 0.6 mL/min and the temperature was 40 °C. The mass spectrometry conditions were electrospray ionization with multi-channel response monitor manner. The test gas pressure: atomized gas 50 psi, heating auxiliary gas 50 psi, curtain gas 35 psi, collision gas 25 psi. Cluster voltage was 80 V, collision voltage was 10 V and collision pool outlet voltage was 17.5 V. Before blood MT concentration test, standard control plasma samples at 1, 2, 10, 30, 100, 300, 1000, 2700 and 3000 ng/mL were prepared by adding to the blank plasma with atorvastatin. The control sample was tested with HPLC-MS/MS as mentioned above and we could get a standard ion peak-concentration curve. Then, through comparing ion peak of MT plasma and standard plasma, the MT plasma concentration could be achieved.

### Data analysis

Electrophysiological data were analyzed and fitted using Clampfit (Axon Instruments, Foster City, CA) and Origin Pro 8 (Origin Lab, USA) software. All data were analyzed by ANOVA or two-tailed student’s *t-*test, and expressed as means ± standard errors of the means (SEM). The statistical significance was set at *P* < 0.05.

## Significance

Chronic pruritus is a disease that is often refractory to the current available medications and seriously compromises the life quality of patients. As a traditional Chinese medicine, *Sophorae Flavescentis Radix (SFR)* has been widely used in treatment of chronic pruritus. To further develop and rationally use *SFR* in the treatment of pruritus patients, the antipruritic mechanism of MT, we studied a major active component of *SFR*. We found that MT had an anti-pruritus effect similar to *SFR* in the mouse models of acute and chronic pruritus. It was further proved that the anti-pruritus effect of MT was by inhibiting the presynaptic N-type calcium channel. These findings would provide an important reference and guidance for the clinical application of MT.

## References

[1] Bautista DM, Wilson SR, Hoon MA (2014). Why we scratch an itch: the molecules, cells and circuits of itch. Nature Neuroscience.

[2] Dustin G, Xinzhong D (2016). The cell biology of acute itch. The Journal of Cell Biology.

[3] Nutten S (2015). Atopic Dermatitis: Global Epidemiology and Risk Factors. Annals of Nutrition & Metabolism.

[4] Caccavale S, Bove D, Bove RM, LA Montagna M (2016). Skin and brain: itch and psychiatric disorders. Giornale italiano di dermatologia e venereologia: organo ufficiale, Societa italiana di dermatologia e sifilografia.

[5] Wong LS, Wu T, Lee CH (2017). Inflammatory and Noninflammatory Itch: Implications in Pathophysiology-Directed Treatments. Int J Mol Sci.

[6] Yosipovitch G, Samuel LS (2010). Neuropathic and psychogenic itch. Dermatologic Therapy.

[7] Buddenkotte J, Steinhoff M (2010). Pathophysiology and therapy of pruritus in allergic and atopic diseases. Allergy.

[8] Lee CH, Yu HS (2011). Biomarkers for itch and disease severity in atopic dermatitis. Current Problems in Dermatology.

[9] Koh WL, Koh MJ, Tay YK (2013). Pityriasis lichenoides in an Asian population. International Journal of Dermatology.

[10] Ogunsanya ME, Kalb SJ, Kabaria A, Chen S (2017). A systematic review of patient-reported outcomes in patients with cutaneous lupus erythematosus. The British journal of dermatology.

[11] Dalgard F, Lien L, Dalen I (2010). Itch in the community: associations with psychosocial factors among adults. Journal of the European Academy of Dermatology & Venereology.

[12] Krishnan A, Koo J (2010). Psyche, opioids, and itch: therapeutic consequences. Dermatologic Therapy.

[13] Oaklander AL, Cohen SP, Raju SV (2002). Intractable postherpetic itch and cutaneous deafferentation after facial shingles. Pain.

[14] Lee SS, Yosipovitch G, Chan YH, Goh CL (2004). Pruritus, pain, and small nerve fiber function in keloids: a controlled study. Journal of the American Academy of Dermatology.

[15] Combs SA, Teixeira JP, Germain MJ (2015). Pruritus in Kidney Disease. Seminars in nephrology.

[16] Kremer AE, Kraus MR (2016). Management of pruritus in patients with cholestatic liver disease. MMW Fortschritte der Medizin.

[17] Liu Q (2009). Sensory neuron-specific GPCR Mrgprs are itch receptors mediating chloroquine-induced pruritus. Cell.

[18] Liu Q (2011). The distinct roles of two GPCRs, MrgprC11 and PAR2, in itch and hyperalgesia. Science Signaling.

[19] Qin L (2012). Mechanisms of Itch Evoked by β-Alanine. Journal of Neuroscience the Official Journal of the Society for Neuroscience.

[20] Mcneil BD (2015). Identification of a mast-cell-specific receptor crucial for pseudo-allergic drug reactions. Nature.

[21] Sun Y-G, Chen Z-F (2007). A gastrin-releasing peptide receptor mediates the itch sensation in the spinal cord. Nature.

[22] Wang CY, Bai XY, Wang CH (2014). Traditional Chinese medicine: a treasured natural resource of anticancer drug research and development. The American journal of Chinese medicine.

[23] Yong J, Wu X, Lu C (2015). Anticancer Advances of Matrine and Its Derivatives. Current pharmaceutical design.

[24] Funaya N, Haginaka J (2012). Matrine- and oxymatrine-imprinted monodisperse polymers prepared by precipitation polymerization and their applications for the selective extraction of matrine-type alkaloids from Sophora flavescens Aiton. Journal of Chromatography A.

[25] Lai JP, He XW, Jiang Y, Chen F (2003). Preparative separation and determination of matrine from the Chinese medicinal plant Sophora flavescens Ait by molecularly imprinted solid-phase extraction. Analytical & Bioanalytical Chemistry.

[26] Sun J (2015). Separation and mechanism elucidation for six structure-like matrine-type alkaloids by micellar liquid chromatography. Journal of Separation Science.

[27] Kamei J (1997). Antinociceptive effects of (+)-matrine in mice. European Journal of Pharmacology.

[28] Kobashi S, Kubo H, Yamauchi T, Higashiyama K (2002). Antinociceptive effects of 1-acyl-4-dialkylaminopiperidine and 1-alkyl-4-dialkylaminopiperidine in mice: structure-activity relation study of matrine-type alkaloids. Biological & Pharmaceutical Bulletin.

[29] Kobashi S, Takizawa M, Kubo H, Yamauchi T, Higashiyama K (2003). Antinociceptive effects of N-acyloctahydropyrido[3,2,1-ij][1,6]naphthyridine in mice: structure-activity relation study of matrine-type alkaloids part II. Biological & Pharmaceutical Bulletin.

[30] Teramoto H (2016). Design and Synthesis of a Piperidinone Scaffold as an Analgesic through Kappa-Opioid Receptor: Structure-Activity Relationship Study of Matrine Alkaloids. Chem Pharm Bull (Tokyo).

[31] Mizuguchi H (2016). Antihistamines suppress upregulation of histidine decarboxylase gene expression with potencies different from their binding affinities for histamine H1 receptor in toluene 2,4-diisocyanate-sensitized rats. J Pharmacol Sci.

[32] Dev S (2009). Kujin suppresses histamine signaling at the transcriptional level in toluene 2,4-diisocyanate-sensitized rats. J Pharmacol Sci.

[33] Yamaguchi-Miyamoto T, Kawasuji T, Kuraishi Y, Suzuki H (2003). Antipruritic effects of Sophora flavescens on acute and chronic itch-related responses in mice. Biol Pharm Bull.

[34] Kini SP (2011). The impact of pruritus on quality of life: the skin equivalent of pain. Archives of dermatology.

[35] Yosipovitch G, Bernhard JD (2013). Clinical practice. Chronic pruritus. The New England journal of medicine.

[36] Zhang JT, Wang W, Duan ZH (2007). Progress of research and application of matrine-type alkaloids. Progress in Modern Biomedicine.

[37] Li T, Wong IKW, Zhou H, Liu L (2010). Matrine Induces Cell Anergy in Human Jurkat T Cells throughModulation of Mitogen-Activated Protein Kinases and Nuclear Factor ofActivated T-Cells Signaling with Concomitant Up-Regulation ofAnergy-Associated Genes Expression. Biological & Pharmaceutical Bulletin.

[38] Liu JY (2007). Effect of matrine on the expression of substance P receptor and inflammatory cytokines production in human skin keratinocytes and fibroblasts. International immunopharmacology.

[39] Todorovic SM, Jevtovic-Todorovic V (2006). The role of T-type calcium channels in peripheral and central pain processing. CNS Neurol Disord Drug Targets.

[40] Zamponi GW, Lewis RJ, Todorovic SM, Arneric SP, Snutch TP (2009). Role of voltage-gated calcium channels in ascending pain pathways. Brain Research Reviews.

[41] Emilie P, Michel V, Jean M, Valerie R (2011). Peptide Neurotoxins that Affect Voltage-Gated Calcium Channels: A Close-Up on ω-Agatoxins. Toxins.

[42] Rahman W, Dickenson AH (2013). Voltage gated sodium and calcium channel blockers for the treatment of chronic inflammatory pain. Neuroscience Letters.

[43] Jarvis SE, Zamponi GW (2001). Interactions between presynaptic Ca2+ channels, cytoplasmic messengers and proteins of the synaptic vesicle release complex. Trends in pharmacological sciences.

[44] Bell TJ, Thaler C, Castiglioni AJ, Helton TD, Lipscombe D (2004). Cell-specific alternative splicing increases calcium channel current density in the pain pathway. Neuron.

[45] Maciel IS (2014). The spinal inhibition of N-type voltage-gated calcium channels selectively prevents scratching behavior in mice. Neuroscience.

[46] Brittain JM (2011). Suppression of inflammatory and neuropathic pain by uncoupling CRMP-2 from the presynaptic Ca²^+^ channel complex. Nature Medicine.

[47] Raingo J, Castiglioni AJ, Lipscombe D (2007). Alternative splicing controls G protein–dependent inhibition of N-type calcium channels in nociceptors. Nature Neuroscience.

[48] Haiyan W (2013). Antinociceptive effects of matrine on neuropathic pain induced by chronic constriction injury. Pharmaceutical Biology.

[49] Gong SS (2016). Neuroprotective Effect of Matrine in Mouse Model of Vincristine-Induced Neuropathic Pain. Neurochemical Research.

[50] Higashiyama K (2005). Implication of the descending dynorphinergic neuron projecting to the spinal cord in the (+)-matrine- and (+)-allomatrine-induced antinociceptive effects. Biological & Pharmaceutical Bulletin.

[51] Xiao P (1999). kappa-Opioid receptor-mediated antinociceptive effects of stereoisomers and derivatives of (+)-matrine in mice. Planta Medica.

[52] Yin LL, Zhu XZ (2005). The involvement of central cholinergic system in (+)-matrine-induced antinociception in mice. Pharmacology Biochemistry & Behavior.

[53] Jain V, Jaggi AS, Singh N (2009). Ameliorative potential of rosiglitazone in tibial and sural nerve transection-induced painful neuropathy in rats. Pharmacological Research.

[54] Thiagarajan VR, Shanmugam P, Krishnan UM, Muthuraman A (2014). Ameliorative effect of Vernonia cinerea in vincristine-induced painful neuropathy in rats. Toxicology & Industrial Health.

[55] Lynch SS, Cheng CM, Yee JL (2006). Intrathecal ziconotide for refractory chronic pain. Annals of Pharmacotherapy.

[56] Williams JA, Day M, Heavner JE (2008). Ziconotide: an update and review. Expert Opin Pharmacother.

[57] Wang, X. & Lin, H. The Clinical Efficacy and Adverse Effects of Interferon Combined with Matrine in Chronic hepatitis B: A Systematic Review and Meta-Analysis. *Phytother Res* **31**, 849–857 (2017). 10.1002/ptr.5808 28382770

[58] Miyamoto T, Nojima H, Shinkado T, Nakahashi T, Kuraishi Y (2002). Itch-associated response induced by experimental dry skin in mice. Jpn J Pharmacol.

